# Nonlinear Mixed Effects Modeling of Glucagon Kinetics in Healthy Subjects

**DOI:** 10.1109/TBME.2023.3262974

**Published:** 2023-08-30

**Authors:** Edoardo Faggionato, Marcello C. Laurenti, Adrian Vella, Chiara Dalla Man

**Affiliations:** Department of Information Engineering, University of Padova, Italy; Division of Endocrinology, Diabetes & Metabolism of the Mayo Clinic College of Medicine, USA. He is now with the Luxembourg Centre for Systems Biomedicine (LCSB), University of Luxembourg, Luxembourg; Division of Endocrinology, Diabetes & Metabolism of the Mayo Clinic College of Medicine, USA; Department of Information Engineering, University of Padova, 35122 Padova, Italy

**Keywords:** Biological system modeling, parameter estimation, diabetes, identification, population modeling

## Abstract

**Objective:**

To date, the lack of a model of glucagon kinetics precluded the possibility of estimating and studying glucagon secretion in vivo, e.g., using deconvolution, as done for other hormones like insulin and C-peptide. Here, we used a nonlinear mixed effects technique to develop a robust population model of glucagon kinetics, able to describe both the typical population kinetics (TPK) and the between-subject variability (BSV), and relate this last to easily measurable subject characteristics.

**Methods:**

Thirty-four models of increasing complexity (variably including covariates and correlations among random effects) were identified on glucagon profiles obtained from 53 healthy subjects, who received a constant infusion of somatostatin to suppress endogenous glucagon production, followed by a continuous infusion of glucagon (65 ng/kg/min). Model selection was performed based on its ability to fit the data, provide precise parameter estimates, and parsimony criteria.

**Results:**

A two-compartment model was the most parsimonious. The model was able to accurately describe both the TPK and the BSV of model parameters as function of body mass and body surface area. Parameters were precisely estimated, with central volume of distribution *V_1_* = 5.46 L and peripheral volume of distribution *V_2_* = 5.51 L. The introduction of covariates resulted in a significant shrinkage of the unexplained BSV and considerably improved the model fit.

**Conclusion:**

We developed a robust population model of glucagon kinetics.

**Significance:**

This model provides a deeper understanding of glucagon kinetics and is usable to estimate glucagon secretion in vivo by deconvolution of plasma glucagon concentration data.

## I. Introduction

IN THE sophisticated physiological control system that regulates glucose homeostasis, glucagon is the principal hormone responsible for the increase of endogenous glucose production to protect against hypoglycemia, which may lead to coma and death. Glucagon is secreted by the α-cells in the pancreatic islets and, due to its hyperglycemic activity, is considered the main antagonist of insulin, which conversely is secreted by the pancreatic β-cells in response to glucose increase to protect from hyperglycemia, whose long-term complication are cardiovascular disease, neuropathy, and nephropathy. A combination of defective insulin secretion and impaired glucagon suppression leads to the development of impaired glucose tolerance in patients with prediabetes [1]. However, while the contribution of pancreatic β-cells has been well studied, the contribution of α-cell dysfunction to the pathogenesis of type 2 diabetes remains understudied in part due to the lack of appropriate methodologies to accurately assess glucagon secretion and action.

To date, the state-of-the-art estimate of insulin secretion is performed by deconvolution of C-peptide plasma concentration employing population parameters of C-peptide kinetics derived from the well-known Van Cauter model [2]. In fact, plasma C-peptide concentration is a better marker of insulin secretion than insulin, since insulin and C-peptide are secreted in a 1:1 ratio, but whereas insulin is extracted by the liver, C-peptide is not. Compared to insulin, the degree of glucagon extraction seems smaller [3] and independent of the glycemic state [4], thus, neglecting the hepatic extraction might be an acceptable hypothesis in the case of glucagon. Therefore, in principle, the same approach used for C-peptide could be used to estimate glucagon appearance, provided that a model of glucagon kinetics is available.

In recent work, we developed a one-compartment model of glucagon kinetics in otherwise healthy individuals [5] and, as before [2], we proposed a model to predict parameters of glucagon kinetics from subject’s body mass index (BMI), sex and body surface area (BSA). This was the first attempt at deriving standardized glucagon kinetic parameters in a medium-sized group of subjects. However, as discussed in [5], the adopted approach suffered from some limitations. For instance, the limited number of samples per subject, coupled with the relatively high lower limit of detectability (LLoD) of the assay, favored a single-compartment to the detriment of a two-compartment model, notwithstanding that the latter performed better in a percentage of the analyzed subjects. This likely provided an estimate of the glucagon volume of distribution (8.2 L on average), which makes it difficult to relate it to the blood volume. Moreover, the two-step procedure (i.e., first, estimating the individual kinetic parameters and, then, calculating the statistical moments) is prone to provide upwardly biased results, as uncertainties on estimated parameters are not considered [6]. However, these drawbacks can be solved by resorting to nonlinear mixed effects (NLME) modeling, which has been proven to improve the quality of the individual parameter estimates and to provide a better description of their between-subject variability in the population with respect to a standard two-stage approach [6]. This allows propagating the uncertainty in the data directly to all the estimates, thus, avoiding biased results. Such approach is widely used in pharmacokinetics-pharmacodynamics studies, but moving from an individual to a population framework has proven to be beneficial also in modeling of glucose-insulin interaction [7], [8] and subcutaneous insulin kinetics [9].

The aims of this work were, first, to test whether a two-compartment model can be identified from the data presented in [5] by switching to the more sophisticated identification framework of NLME models and, second, to incorporate covariates into the model, so that the coefficients linking them to the kinetic parameters are treated as other model parameters and are estimated directly from the data.

## II. Methods

### A. Database

The database is the same as used in [5] and consists of glucagon pharmacokinetic data gathered from 53 healthy subjects (36 females and 17 males, age = 54.5 ± 12.5 years, weight = 81.3 ± 15.3 kg). Common demographic and anthropometric characteristics of the subjects, hereafter referred as covariates, were also available for the analysis (a summary is reported in Table I).

The study was performed after the approval of the Mayo Institutional Review Board (Mayo Clinic College of Medicine, Rochester, MN, September 15, 2017, protocol no. 17-006493). Volunteers received a primed infusion of labeled [3-^3^H] glucose (10 μCi prime and 0.1 μCi/min continuous) from −180 minutes until the start of the experiment (t = 0 min), when a ‘prandial’ labeled [3-^3^H] glucose infusion was administered to reproduce an oral ingestion of 75 g of glucose. Simultaneously, an infusion of somatostatin (60 ng/kg/min) to inhibit endogenous glucagon and insulin secretion, and a variable infusion of insulin to replace normal insulin levels were started and lasted till the end of the experiment (t = 300 min). Finally, from t = 120 min to the end of the experiment, exogenous glucagon was infused at a constant rate (65 ng/kg/min).

**Fig. 1.**
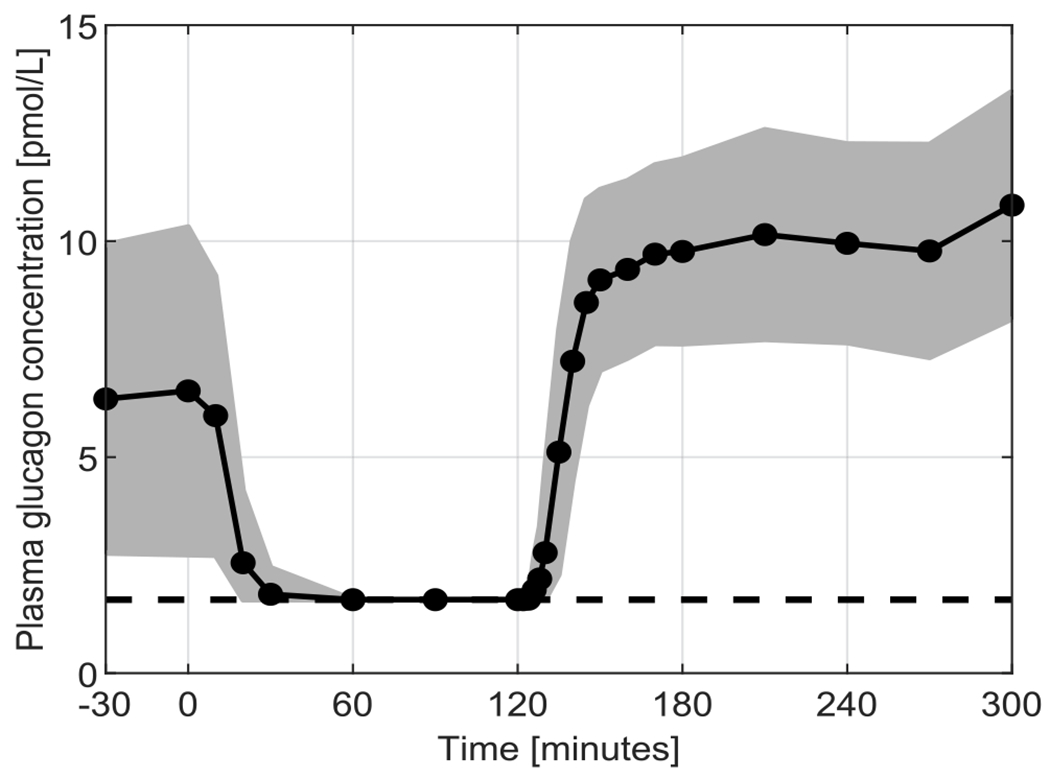
Mean (black solid line) ± standard deviation (grey area) of the measured glucagon concentration during the experiment. Black dots indicate sampling times. Black dashed line represents LLoD. The figure differs from Fig. 1 in [5], since there data below LLoD were considered zero.

Blood samples were collected at t = [−30 010 20 30 60 90 120 122 124 126 128 130 135 140 145 150 160 170 180 210 240 270 300] min after the start of the experiment to measure plasma glucagon concentration using a two-site ELISA (Mercodia, Winston Salem, NC). The essay had a LLoD of 1.7 pmol/L and an upper limit of detectability of 130 pmol/L. Mean time course and standard deviation of measured glucagon concentration are reported in Fig. 1. More detailed information about the experimental protocol is available in [5].

### B. Models

The analysis of glucagon kinetics was conducted within a NLME estimation framework. This approach provides estimates of the so-called fixed effects, which comprises typical population values of the kinetic parameters together with the covariate contributions to each kinetic parameter, and the so-called random effects, which describe the between- and within-subject variability of model parameters.

A NLME model consists in a hierarchy of models, each one aiming to describe the various levels of variability present in the data. For the purposes of this work, a two-level hierarchy was considered: the individual subject response, modeled through a structural kinetic model, and the overall population variability, modelled through a stochastic model. The remaining variability was considered as unexplained residual noise, modeled though an error model.

#### Structural model:

**Fig. 2.**
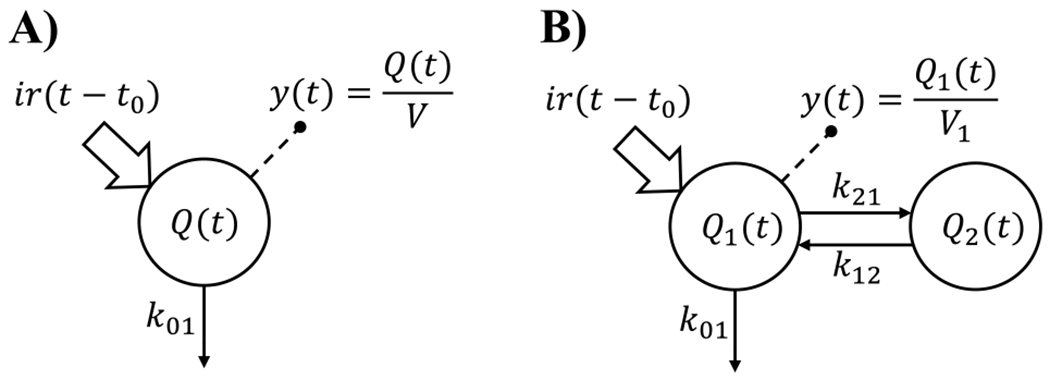
Compartmental representation of the two tested kinetic models. *Panel A*: Delayed one-compartment model. *Panel B*: Delayed two-compartment model. Signals ir and y represent constant infusion of glucagon and measurements of plasma hormone concentration, respectively. $Q,Q_{1}$, and $Q_{2}$ represent the amount of glucagon in their respective compartment.

Similarly to what we did previously [5], we tested both a delayed one-compartment (Fig. 2, Panel A) and a delayed two-compartment model (Fig. 2, Panel B).

The differential equations of the one-compartment model are:

(1) $$\{\begin{array}{l} \dot{Q}\;(t)=-k_{01}Q\;(t)+ir\;\left(t-t_{0}\right)\\ y\;(t)=\frac{Q(t)}{V} \end{array}$$

where Q(t) (pmol) is the amount of glucagon in the system, $k_{01}$ (min^−1^) is the glucagon fractional clearance rate, ir (pmol/min) is the infusion rate of the pump, $t_{0}$ (min) is a zero-order delay from the start of glucagon infusion, y (pmol/L) is the plasma glucagon concentration and *V* (L) is the glucagon volume of distribution.

The differential equations for the two-compartment model are:

(2) $$\{\begin{array}{l} {\dot{Q}}_{1}\;(t)=-\left(k_{01}+k_{21}\right)\;Q_{1}\;(t)+k_{12}Q_{2}\;(t)+ir\;\left(t-t_{0}\right)\\ {\dot{Q}}_{2}\;(t)=-k_{12}Q_{2}\;(t)+k_{21}Q_{1}\;(t)\\ y\;(t)=\frac{Q_{1}(t)}{V_{1}} \end{array}$$

where $Q_{1}(t)$ and $Q_{2}(t)$ are the amounts of glucagon in the central and peripheral compartment, respectively, $k_{12}$ and $k_{21}$ (min^−1^) are the between-compartment transfer rates, and $V_{1}$ and $V_{2}$ (L) are the glucagon volumes of distribution in the central and peripheral compartment, respectively.

The models were then reparametrized, to explicit the clearance rate and volume instead of the fractional rates. This reparameterization allows for a more physiological interpretation of model parameters, as it separates the effect of the organs responsible for the elimination of the hormone from the volume of distribution. This may introduce minor differences when identifying the model at an individual level, but it becomes crucial in a population framework, where systems are not deterministic and parameters are random variables [10]. In addition, constant rates measured in *time^−1^* blend the functionality of the organ and body size effects in one parameter, making the introduction of physiological and pathophysiological characteristics into the model more challenging. The equations to switch from one formulation to the other are:

(3) $$CL=V_{1}k_{01}$$

(4) $$BCL=V_{1}\;k_{21}=V_{2}k_{12}$$

where CL (L/min) is the glucagon clearance rate, BCL (L/min) is the between-compartment clearance and $V_{2}$ (L) is the volume of distribution of the peripheral compartment.

The time lag, $t_{0}$, was introduced to account for the delay elapsed from the activation of the pump to the time it takes for glucagon to overcome the threshold of detectability. This lag was due to three main contributions: first, the mechanical friction of the pump in pushing the solution with glucagon through the length of the cannula to the vein (~5 minutes), second, the transport of glucagon from the site of injection to the sampling artery (~2 minutes) and, finally, the delay due to the sparse sampling schedule after t = 130 min, which, considering the relatively high LLoD, might considerably delay the detection of the first sample above the LLoD. Neglecting $t_{0}$ in the model, would have led to a significant over-estimation of V and $V_{1}$ (not shown).

#### Stochastic model:

The variability of kinetic parameters was described through a stochastic model assuming that each parameter follows a log-normal distribution:

(5) $$\psi_{i,j}=\theta_{i}\;\text{exp}\;\left(\eta_{i,j}\right)$$

where $\psi_{i,j}$ is the *i^th^* individual parameter of the *j^th^* subject, $\theta_{i}$ is the *i^th^* population parameter (the so-called fixed effects) and $\eta_{i,j}$ are Gaussian random variables η~N(0,Ω) (the so-called random effects). The covariance matrix, Ω, includes diagonal elements, $\omega_{n}^{2}$, representing the variance of the *n^th^* random effect but may also include out-of-the-diagonal elements, $\rho_{mn}\omega_{m}\omega_{n}$, where $\rho_{mn}$ represents the correlation between the *m^th^* and the *n^th^* random effect.

Moreover, covariate effects can also be included in the stochastic model. In this case, the model of the individual parameter becomes:

(6) $$\psi_{i,j}=\theta_{i}\;\text{exp}\;\left[\beta_{i,k}\;\text{log}\;\left(\frac{c_{j,k}}{\bar{c}}\right)+\eta_{i,j}\right]$$

where the coefficient $\beta_{i,k}$ quantifies how much a deviation of the individual covariate, $c_{j,k}$, from the median in the population, $\overset{‾}{c}$, translates into a variation of the individual parameter $\psi_{i}$ from the estimated fixed effect $\theta_{i}$.

#### Error model:

The residual unexplained variability was assumed to be an additive Gaussian random noise with standard deviation that is the sum of two components, one constant, *a*, and one proportional, through the parameter *b*, to the measured concentration, y(t).

Here, parameters *a* and *b* are fixed to 0.07 pmol/L and 0.049 , respectively, that were determined experimentally by the quality control of the Mayo Clinic laboratory.

### C. Allometric Scaling

Allometric models describe the dependences of common pharmacokinetic parameters on measurable body size metrics. These models are supported by physiological considerations and fractal geometric concepts and confirmed by observations in different biological fields [11]. The most common body size relation is a power function of the form:

(7) $$\psi_{j}=\alpha \cdot {\left(\frac{W_{j}}{\overset{‾}{W}}\right)}^{\beta }$$

where $\psi_{j}$ is the individual physiological parameter of the *j^th^* subject, $W_{j}$ is the individual body size (e.g., body weight), $\overset{‾}{W}$ is the mean body size in the population, and α and β are empirical coefficients. Normalization by $\overset{‾}{W}$ is introduced to avoid bias in the estimation of the fixed effect α. Usually, when applied to physiological volumes, such as V, this relationship is linear, β=1, whereas for metabolic rates, such as CL, it is a power function with β=3/4 [11].

The use of allometric scaling allows describing a fraction of the parameter inter-subject variability in a deterministic fashion employing easily accessible metrics. Therefore, in principle, allometric scaling can be employed to improve the performance of the model without the need of estimating additional parameters.

In this work, we used allometric relationship in the stochastic models of glucagon volume of distributions, $V,V_{1}$ and $V_{2}$, and of glucagon clearances, CL and BCL, so that:

(8) $$V_{j}=V^{\text{pop}}\;\left(\frac{W_{j}}{\overset{‾}{W}}\right)\;\text{exp}\;\left(\eta_{V,j}\right)$$

(9) $$CL_{j}=CL^{\text{pop}}\;{\left(\frac{W_{j}}{\overset{‾}{W}}\right)}^{3/4}\;\text{exp}\;\left(\eta_{CL,j}\right)$$

where $V^{pop}$ and $CL^{pop}$ are the fixed effects and $\eta_{V,j}$ and $\eta_{CL,j}$ the random effects associated to relative kinetic parameters.

### D. Model Identification

For model identification, we employed the software Monolix (version 2020R1, ©Lixoft, Antony, France [12]), which implements the Stochastic Approximation of the Expectation Maximization (SAEM) algorithm in combination with the Metropolis-Hastings algorithm to estimate the parameters that maximize the likelihood of the data. When possible, estimates of the kinetic parameters were initialized to the mean values obtained in [5]. $V_{1}^{\text{pop}}$ and $V_{2}^{\text{pop}}$ in the two-compartment model were both initialized to a reasonable value of 5 L, as no information was available for those parameters in the literature. Initial values of ω and ρ were set to 1 and 0, respectively. *A priori* information obtained from [5] was employed for the estimation of the time lag, $t_{0}^{pop}$. Models were identified only on data collected from 120 min onward, when one can safely assume that the endogenous glucagon is absent due to the somatostatin infusion.

It is worth noting that, in a NLME model identification, data below the LLoD can be taken into account in estimating model parameters [12]. In fact, Monolix, implements an extension of the SAEM algorithm able to compute the likelihood also for censored data [13]. Therefore, such information is efficiently used in the estimation of model parameters. This is not the case when one resorts to an individual weighted least-squares approach, in which data below the LLoD are often not weighted in the analysis, thus leading to biased results.

### E. Model Selection

The best NLME model of glucagon kinetics was selected using a three-stage procedure.

First, we compared the one-compartment and the two-compartment structures. In this first stage, the covariance matrix Ω was assumed to be diagonal and no covariates were included in the models. See Table II-A in the Results.

Second, the covariance matrix of the best model was populated by forward inclusion of correlation parameters $\left(\rho_{V_{1},CL}\right.,\;\rho_{V_{1},V_{2}}$, etc.) followed by a backward elimination to select only the stable features. However, *a priori* we excluded any correlation between the time lag, $t_{0}$, and the other model parameters, since $t_{0}$ is related to the experimental set-up and not to physiological processes. At this stage, ten models were evaluated in total, one is the model with only diagonal elements in the Ω matrix, six including only one out-of-diagonal element, and three including a new combination of out-of-diagonal elements (all the possible combinations allowed by the software, which accepts only full blocks of correlations in Ω [12]). The model with full Ω was not tested since model performance did not improve in the previous step. See Table II-B in the Results.

Finally, allometric scaling was applied to the model with the best covariance matrix to find the best body size descriptor for each kinetic parameter. As before, the adopted strategy used a forward inclusion followed by a backward elimination. Since the available body size descriptors were highly correlated one to each other, no more than one covariate per kinetic parameter was employed in each tested model. As in the previous stage, we *a priori* excluded any possible covariate effect on the lag, $t_{0}$. Twenty-four models were evaluated in total, one is the model without any covariate effect, twenty are models associating one kinetic parameter $\left(CL,V_{1},BCL\right.$ and $\left.V_{2}\right)$ to one of the available covariates (total body mass, TBM, lean body mass, LBM, height, H, body surface area, BSA, body mass index, BMI), keeping at each step the most significant covariate-parameter pair, and three are the models were we tried to remove the selected pairs (backward elimination). See Table II-C in the Results.

At each stage, the tested models were compared in terms of physiological plausibility of the estimates, ability to fit the data by inspection of the residual distribution, and precision of estimates by computing the relative standard error (RSE).

In particular, the goodness of residuals was assessed by visual inspection of the individual weighted residual (IWRES) distribution and of the normalized prediction distribution errors (NPDE), a non-parametric version of the population weighted residual distribution [14]. A final overview of model predictive ability was summarized with the so-called visual predictive check (VPC) plot, which compares the percentiles of data obtained from multiple Markov chain Monte Carlo simulations with the empirical percentiles of the observed data. Since glucagon infusion was normalized to the LBM of the subject, the prediction-corrected VPC (pcVPC) plot was used [15].

Models that performed satisfactorily in terms of previous metrics were compared using a Bayesian information criterion corrected for NLME models (BICc) [16], and finally, the model that scored the lowest BICc was selected as best one.

## III. Results

### A. Structural Model Assessment

Results obtained with both the one- and the two-compartment models were satisfactory in terms of residual distribution and precision of the estimated parameters, with the one-compartment model providing the most precise parameter estimates and the two-compartment providing the highest likelihood (Table II-A). The parsimony criteria suggested the selection of the two-compartment model since it scored a lower value of the BICc (3272.22) compared to the simpler one-compartment model (3535.96).

### B. Covariance Model Assessment

According to the forward inclusion and backward elimination algorithm, models including two or more correlation terms did not perform better than models including just one correlation term. All the six models including one correlation term provided good residual distribution and precision of the estimates, whereas models with more than one correlation term presented at least one parameter estimated with RSE>100%. Among the six former models, the model providing the lowest BICc (3252.76) was the one including the correlation between CL and V_1_, $\rho_{CL,V_{1}}=0.72$ (Table II-B). The addition of $\rho_{CL,V_{C1}}$ did not significantly change any of the estimated parameters, except V_2_ that dropped from a value of 8.72 L to a value of 5.47 L after the inclusion of such a correlation term. It is worth noting that this was also the parameter estimated with less precision (RSE = 44% and 39%, before and after including the correlation, respectively).

### C. Allometric Model Assessment

All the tested models provided good residual distribution and precision of the estimates. Among them, the model providing the lowest BICc (3234.77) was the one employing TBM for the allometric scaling of $V_{1}$, and BSA for $CL,V_{2}$, and BCL (Table II-C).

Allometric scaling did not significantly change any of the model parameter estimates.

### D. Final Model

The equations of the final NLME model are reported in (10) and (11), shown at the bottom of the next page.


(10) $$\psi :\{\begin{array}{c} CL=CL^{pop}\cdot {\left(\frac{\text{BSA}}{\bar{\text{BSA}}}\right)}^{\frac{3}{4}}\cdot \text{exp}\;\left(\eta_{CL}\right)\\ V_{1}=V_{1}^{pop}\cdot \left(\frac{\text{TBM}}{\bar{\text{TBM}}}\right)\cdot \text{exp}\;\left(\eta_{V_{1}}\right)\\ BCL=BCL^{pop}\cdot {\left(\frac{\text{BSA}}{\bar{\text{BSA}}}\right)}^{\frac{3}{4}}\cdot \text{exp}\;\left(\eta_{BCL}\right)\\ \begin{array}{l} V_{2}=V_{2}^{pop}\cdot \left(\frac{\text{BSA}}{\bar{\text{BSA}}}\right)\cdot \text{exp}\;\left(\eta_{V_{2}}\right)\\ t_{0}=t_{0}^{pop}\cdot \exp\;\left(\eta_{t_{0}}\right) \end{array} \end{array}$$

**Fig. 3.**
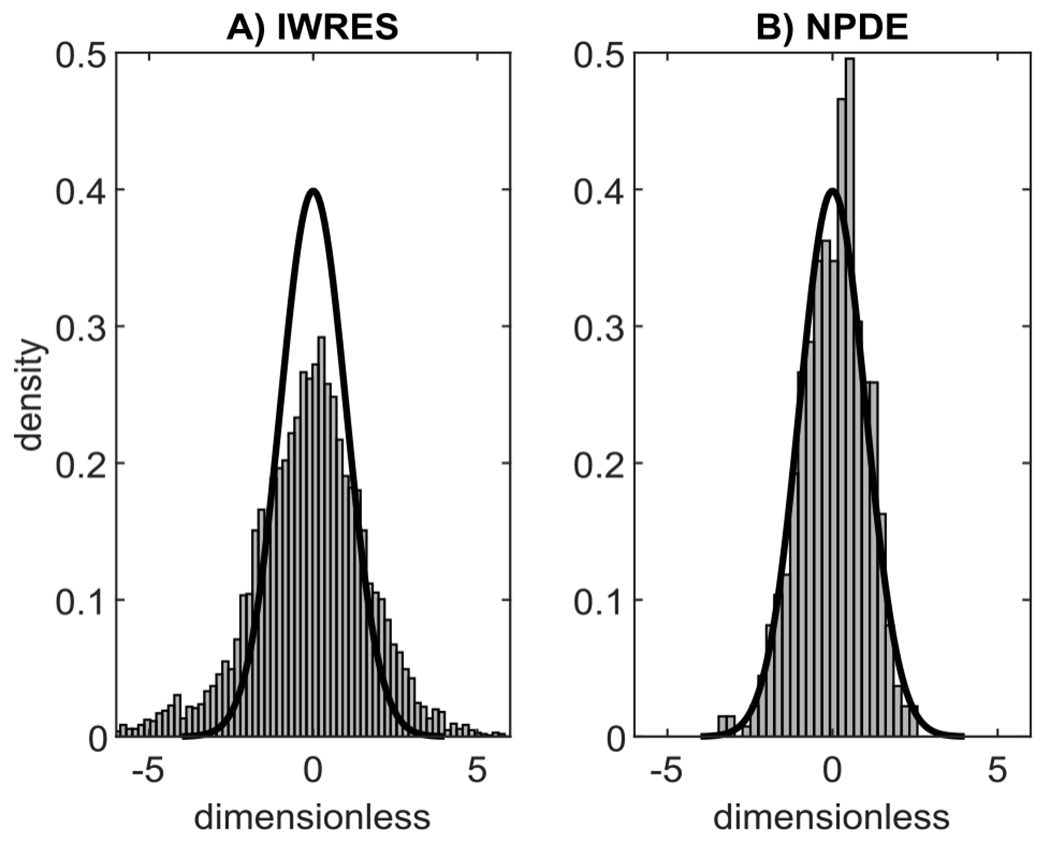
Evaluation of the goodness of residuals of the best model. *Panel A*: Empirical distributions of IWRES (grey bars) compared with a standard Gaussian distribution (black line). *Panel B*: Empirical distributions of NPDE (grey bars) compared with a standard Gaussian distribution (black line).

**Fig. 4.**
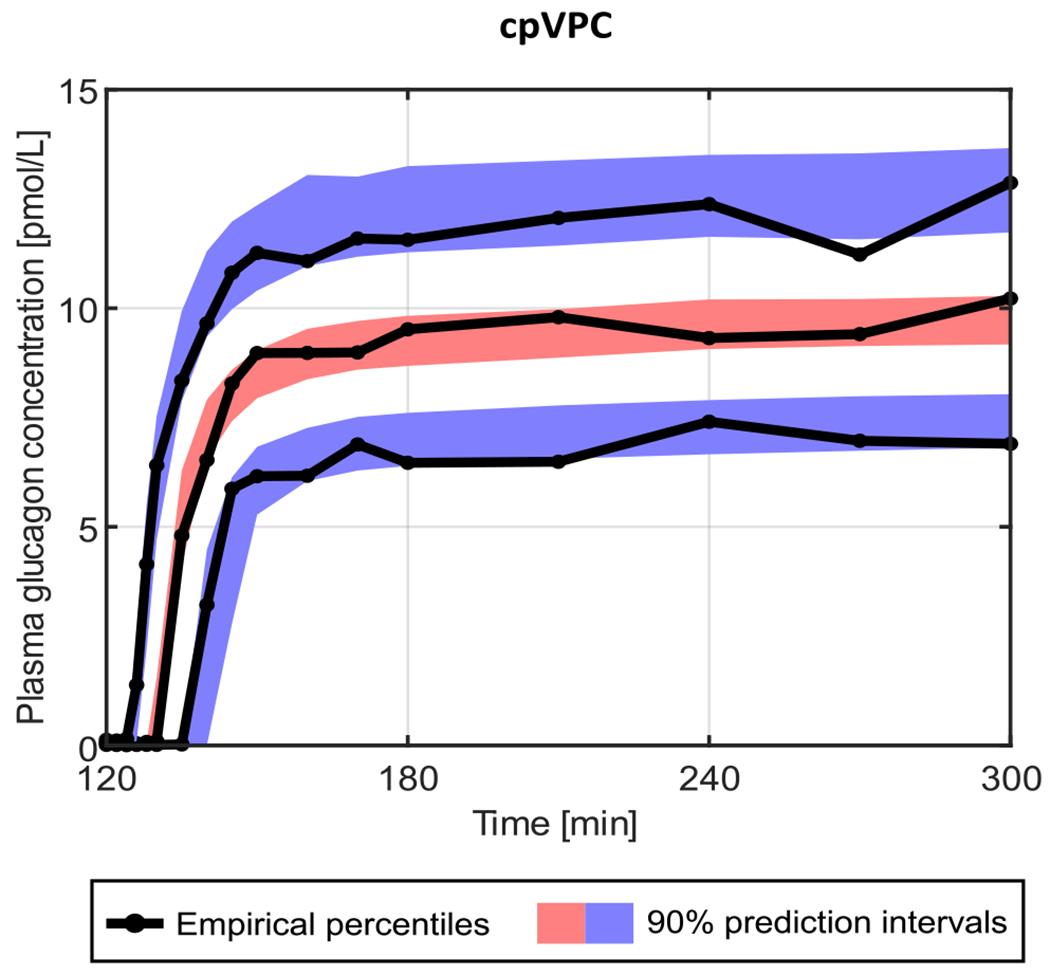
Visual predictive check obtained with the final model. The 90% prediction intervals of the 10th (blue lower area), 50th (red central area), and 90th (blue upper area) percentiles are compared with the 10th, 50th, and 90th empirical percentiles (black solid lines).

All population parameters were within physiological ranges and both CL and $t_{0}$ were in agreement with that estimated in [5]. The estimated parameters are reported in Table III together with their precision. IWRES and NPDE are reported in Panel A and B of Fig. 3, respectively. The pcVPC plot is shown in Fig. 4.

The model in (10) can be used to predict the kinetic parameters of a subject without performing any experiment, by simply fixing the random effects, η, to their expected value (zero in this case).

## IV. Discussion

In this work, we developed a robust two-compartment NLME model of glucagon kinetics, which overcame most of the limitations of previously proposed models, including ours [5]. Moving from an individual two-stage modeling to a NLME population approach, we found that a two-compartment model was more parsimonious than a single-compartment model to describe glucagon kinetics. Indeed, working in a NLME framework and using the SAEM algorithm to estimate the parameters that maximize the likelihood of the data, we could exploit the information that some measurement was below the level of detectability (LLoD), instead of simply discarding those samples. This, in turn, allowed inclusion in the analysis of 2 of the 53 subjects of the original data set that were discarded in [5]. In fact, in those subjects, glucagon kinetics was so rapid that the first glucagon sample above the LLoD had reached the steady-state concentration, making it impossible to estimate the distribution volume using a single-individual approach. Conversely, in a NLME framework, the population estimates and the covariates are used to help individual estimation of the kinetic parameters, making this possible even in data-poor conditions. Indeed, population approaches are based on the assumption that all subjects are random realizations of the population and they share some typical features common to the population itself (fixed effects).


(11) $$\Omega =\left[\begin{array}{ccccc} \omega_{CL}^{2} & \rho_{CL,V_{1}}\omega_{CL}\omega_{V_{1}} & 0 & 0 & 0\\ \rho_{CL,V_{1}}\omega_{CL}\omega_{V_{1}} & \omega_{V_{1}}^{2} & 0 & 0 & 0\\ 0 & 0 & \omega_{BCL}^{2} & 0 & 0\\ 0 & 0 & 0 & \omega_{V_{2}}^{2} & 0\\ 0 & 0 & 0 & 0 & \omega_{t_{0}}^{2} \end{array}\right]$$

The population information is then employed to support the individual parameter estimation process, because each subject is characterized by random effects, which represent the deviation from the typical behavior of the population.

Model ability to fit the data was good at both individual and population levels (Fig. 3), even if the distribution of the IWRES showed two symmetric heavy tails, suggesting that the unexplained residual variability was not entirely attributable to instrument error noise. Indeed, we assumed that the residual unexplained variability was an additive Gaussian random noise with standard deviation linearly dependent on the model-predicted glucagon concentration, whose driving coefficients were fixed to values determined experimentally by repeated measures. However, other factors, besides instrument error noise, may affect glucagon concentration data and contribute to data variability, e.g., the possible discrepancy between the scheduled and the actual sampling time, which is usually negligible but may be not here, since glucagon kinetics are very rapid. Therefore, we tried an *a posteriori* estimate of such coefficients. However, this led to a distribution of the IWRES that was too skewed to the center (not shown). We concluded that, likely, the best values of such coefficients were somewhere in between the experimentally derived and the *a posteriori* estimate.

All estimated parameters were physiologically plausible and, overall, in line with the established literature reporting results of intravenous experiments [5], [17], [18], [19], [20], except for the volume of distribution, whose estimate might be affected by the experimental protocol. Of note, here, the volume of distribution of the central compartment, $V_{1}$, was 5.46 L and that of the peripheral compartment, $V_{2}$, 5.51 L. The former value was very close to the plasma volume of an adult individual, whereas the latter can be interpreted as the extravascular space where the glucagon distributes. Conversely, the one-compartment model presented in [5] and [20] provided a much higher value of the volume of distribution, which made the single compartment difficult to interpret as a physical space. Comparable results would have been obtained even with the NLME approach if a single-compartment representation were assumed (7.23 L).

Glucagon clearance estimated in this work compared well with that reported in studies that modeled glucagon kinetics after subcutaneous hormone administration [21], [22]. This was not the case for volume of distribution. However, the estimate of this parameter is strongly dependent on model order (e.g., one or two compartments) and different experiments, which do or do not suppress endogenous glucagon secretion and use either the intravenous or the subcutaneous route of administration (in particular, with the latter, glucagon bioavailability may be lower than 100%).

The parameter estimated with the poorest, despite acceptable, precision was $V_{2}$ (RSE = 42%), and this also presented the highest variability among subjects. Interestingly, this parameter was the one estimated with the poorest precision also in [5], making the authors prefer the one-compartment structure. We hypothesized that the inability to estimate this parameter with precision was due, at least in part, to the experimental protocol design, with a constant infusion of exogenous glucagon (after the somatostatin-induced suppression of endogenous glucagon secretion). To test this hypothesis, we used the model to simulate 53 glucagon profiles either after a bolus, a constant infusion, or a primed-continuous infusion (i.e., a bolus followed by a constant infusion) of glucagon, using the same sampling schedule and the same LLoD of the present experiment. This analysis confirmed that accounting for the data below the LLoD made the constant infusion adequate to estimate $V_{2}$, but results could have significantly improved if one would have used a primed-continuous infusion (RSE of $V_{2}$ dropped to 29%). However, using a primed-continuous infusion of glucagon may not be feasible in practice, since rapidly injecting a large amount of glucagon into the circulation is likely to cause nausea and stimulate insulin secretion.

Another advantage of using NLME modeling is the possibility to examine the causes underlying the variability present in the data and identify whether some independently assessed characteristics of the subjects, normally referred to as covariates, significantly correlate with the model parameter values. These features can be integrated into the population model itself to improve its predictive power. The coefficients driving the relationships between the individual parameter values and the covariates can, in fact, be introduced in the model as additional parameters and therefore optimized together with the remaining population fixed effects. In this way, a part of the population variability is explained in a deterministic fashion, rather than by means of individual random effects.

Here, part of the variability present in the system was described by scaling model parameters by some body size descriptors in accordance with allometric relationships. In this way, it was possible to introduce covariates into the model without undermining estimates precision. The introduction of covariates through allometric scaling led to a significant shrinkage of the between-subject variability for parameters $CL,V_{1}$ and BCL compared to the model that did not employ covariates (with a drop of their coefficient of variation of 5%, 2% and 6%, respectively). In particular, BSA and TBM were good predictors of model parameters. It is worth noting that these covariates do not require sophisticated instrumentation to be obtained (such as an X-ray scanner to measure LBM), making the model usable by most of the investigators.

We also tried to incorporate age and gender into the model. However, unlike other anthropometric descriptors, we could not exploit allometric scaling and had to estimate one additional parameter for each introduced covariate. This resulted in an unacceptable degradation of the precision of the parameter estimates, which resulted in their exclusion as covariates.

In this work, glucagon kinetics were studied in the absence of endogenous glucagon secretion thanks to the infusion of somatostatin. Whether or not this hormone can affect glucagon kinetics remains an open question. Another possible limitation of this work is that the data base consisted of a limited number of quite homogeneous subjects (53 healthy subjects, mainly females and slightly overweight). Including more subjects with a wider span of covariates and pathological statuses would have allowed development of a more comprehensive model providing a deeper insight into the kinetics of glucagon. However, at least in regard to glucagon clearance, a recent work reported that this parameter was preserved in patients with type 2 diabetes [20]. In the absence of a suitable external dataset, we should note that this population model was only validated on data used for the identification.

Future work includes the extension of this model to other populations, such as younger or older subjects, with type 1 or type 2 diabetes, the investigation of other covariates as descriptors for model parameters, such as pathological statuses, possible medications, or different genotypes, and the assessment of model performance on an independent dataset.

## V. Conclusion

In this work, a two-compartment model describing glucagon kinetics in plasma has been developed in an NLME framework. The model accurately predicted the glucagon profiles after an intravenous continuous infusion, provided a robust estimate of the between-subject variability that affects glucagon kinetics parameters, employing also easily accessible subject covariates.

We believe that this will allow a step forward towards a better understanding of glucagon secretion in humans, since such a model can be used to estimate glucagon secretion via deconvolution, as currently done for insulin. That would be of great value since glucagon secretion and action and their role in the pathogenesis of diabetes have been understudied, compared to insulin, also due to the lack of appropriate methodologies to accurately assess them. Finally, once validated in subjects with type 1 diabetes, this model can be incorporated into diabetes simulation platforms to assess the efficacy of dual-hormone artificial pancreas.

## Figures and Tables

**TABLE I:** Summary of the Covariates

Gender	36 / 37	females / males
Age, years	54.5 ± 12.5	years
Total body mass (TBM)	81.3 ± 15.3	kg
Lean body mass (LBM)	47.1 ± 10.4	kg
Height (H)	170 ± 9.8	cm
Body mass index (BMI)	28.2 ± 3.9	kg/m^2^
Body surface area (BSA)	1.92 ± 0.22	m^2^

Data are reported as mean ± standard deviation.

**TABLE II-A:** Comparison Between Structural Models

Model	$\bar{RSE}$	−2LL	BICc
one compartment	7.87	3503.67	3535.96
**two compartments**	**14.34**	**3218.40**	**3272.22**

Summary of the performance of the two evaluated models used for the structural model selection. The average residual standard error $\left(\bar{RSE}\right)$, the log-likelihood ratio (−2LL) and the corrected Bayesian information criteria (BICc) are reported. The best model is highlighted in bold.

**TABLE II-B:** Comparison Among Stochastic Models

Model	$\bar{RSE}$	−2LL	BICc
two compartments with no correlation	14.34	3218.40	3272.22

$\rho_{CL,V_{1}}$	**14.63**	**3194.97**	**3252.76**
$\rho_{CL,BCL}$	12.72	3204.49	3262.28
$\rho_{CL,V_{2}}$	15.45	3212.07	3269.85
$\rho_{V_{1},BCL}$	15.50	3207.98	3265.76
$\rho_{V_{1},V_{2}}$	13.07	3216.30	3274.08
$\rho_{BCL,V_{2}}$	14.03	3239.71	3297.49

$\rho_{CL,V_{1}}\;\rho_{CL,V_{2}}\;\rho_{V_{1},V_{2}}$	71.23	3192.14	3257.86
$\rho_{CL,V_{1}}\;\rho_{CL,BCL}\;\rho_{V_{1},BCL}$	24.05	3192.42	3258.14
$\rho_{CL,V_{1}}\;\rho_{BCL,V_{2}}$	34.59	3195.60	3257.35

Summary of the performance of the ten evaluated models used for the stochastic model selection. The log-likelihood ratio (−2LL), the average residual standard error $\left(\bar{RSE}\right)$ and the corrected Bayesian information criteria (BICc) are reported. The best model is highlighted in bold.

**TABLE II-C:** Comparison Among Models Including Covariates

Model description	$\bar{RSE}$	−2LL	BICc
two compartments with $\rho_{CL,V_{1}}$	14.63	3194.97	3252.76

$\beta_{CL,TBM}$	16.57	3193.73	3251.51
$\beta_{CL,LBM}$	16.35	3198.08	3255.86
$\beta_{CL,H}$	13.57	3189.72	3247.51
$\beta_{CL,BSA}$	14.25	3186.58	3244.36
$\beta_{CL,BMI}$	14.99	3201.35	3259.13

$\beta_{CL,BSA}\;\beta_{V_{1,}TMB}$	16.60	3180.66	3238.44
$\beta_{CL,BSA}\;\beta_{V_{1,}LBM}$	13.66	3185.73	3243.52
$\beta_{CL,BSA}\;\beta_{V_{1,}H}$	14.09	3184.61	3242.40
$\beta_{CL,BSA}\;\beta_{V_{1,}BSA}$	16.43	3181.44	3239.23
$\beta_{CL,BSA}\;\beta_{V_{1,}BMI}$	13.21	3181.72	3239.50

$\beta_{CL,BSA}\;\beta_{V_{1},TBM}\;\beta_{V_{2},TBM}$	13.94	3179.66	3237.44
$\beta_{CL,BSA}\;\beta_{V_{1},TBM}\;\beta_{V_{2},LBM}$	17.18	3179.60	3237.39
$\beta_{CL,BSA}\;\beta_{V_{1},TBM}\;\beta_{V_{2},H}$	13.81	3181.24	3239.02
$\beta_{CL,BSA}\;\beta_{V_{1},TBM}\;\beta_{V_{2},BSA}$	14.43	3178.36	3236.14
$\beta_{CL,BSA}\;\beta_{V_{1},TBM}\;\beta_{V_{2},BMI}$	15.14	3180.20	3237.99

$\beta_{CL,BSA}\;\beta_{V_{1},TBM}\;\beta_{V_{2},BSA}\;\beta_{BCL,TBM}$	14.46	3178.93	3236.72
$\beta_{CL,BSA}\;\beta_{V_{1},TBM}\;\beta_{V_{2},BSA}\;\beta_{BCL,LBM}$	15.13	3180.15	3237.94
$\beta_{CL,BSA}\;\beta_{V_{1},TBM}\;\beta_{V_{2},BSA}\;\beta_{BCL,H}$	14.72	3188.84	3246.62
$\beta_{\mathrm{CL},\mathrm{BSA}}\;\beta_{V_{1},\mathrm{TBM}}\;\beta_{V_{2},\mathrm{BSA}}\;\beta_{\mathrm{BCL},\mathrm{BSA}}$	**13.74**	**3176.98**	**3234.77**
$\beta_{CL,BSA}\;\beta_{V_{1},TBM}\;\beta_{V_{2},BSA}\;\beta_{BCL,BMI}$	12.93	3179.65	3237.43

$\beta_{V_{1},TBM}\;\beta_{V_{2},BSA}\;\beta_{BCL,BSA}$	15.03	3198.36	3256.14
$\beta_{CL,BSA}\;\beta_{V_{2},BSA}\;\beta_{BCL,BSA}$	15.56	3182.61	3240.39
$\beta_{CL,BSA}\;\beta_{V_{1},TBM}\;\beta_{BCL,BSA}$	14.21	3182.09	3239.87

Summary of the performance of the twenty-four evaluated models used for the covariate model selection. The log-likelihood ratio (−2LL), the average residual standard error ($\bar{RSE})$, and the corrected Bayesian information criteria (BICc) are reported. The best model is highlighted in bold.

**TABLE III:** Estimated Model Parameters

	Parameter	Estimated Value	Unit	RSE [%]
Fixed effects	$CL^{\text{pop}}$	1.25	L/min	3
	$V_{1}^{\text{pop}}$	5.46	L	7
	$BCL^{\text{pop}}$	0.42	L/min	12
	$V_{2}^{\text{pop}}$	5.51	L	42
	$t_{0}^{\text{pop}}$	10.55	min	7

Standard deviation of the random effects	$\omega_{CL}$	0.19	dimensionless	13
	$\omega_{V_{1}}$	0.38	dimensionless	14
	$\omega_{BCL}$	0.49	dimensionless	17
	$\omega_{V_{2}}$	2.46	dimensionless	15
	$\omega_{t_{0}}$	0.48	dimensionless	10

Correlations	$\rho_{CL,V_{1}}$	0.75	dimensionless	14

Estimates of model parameters together with their precision expressed as relative standard error (RSE).
